# Use of sevoflurane in the medical ICU: 2-year experience, patient and safety profile

**DOI:** 10.1186/cc14574

**Published:** 2015-03-16

**Authors:** A Koroša, A Markota, F Svenšek, A Sinkovič

**Affiliations:** 1 University of Maribor, Slovenia; 2 University Medical Centre Maribor, Slovenia

## Introduction

The aim of this study is to present our experience with sevoflurane in the ICU, outline which patients were sedated with sevoflurane and present the safety profile. Sevoflurane has some potential advantages over intravenous sedation: rapid elimination and few interactions. The optimal role of sevoflurane in ICU is not known.

## Methods

We performed a retrospective study on adult patients who were sedated with sevoflurane in the medical ICU. The decision to use sevoflurane was left to the attending physician. Institutional ethics committee approval was obtained. The target mean alveolar concentration in all patients was 0.5 to 1%. The AnaConDa® device (Sedana Medical, Uppsala, Sweden) was used along with the Anastasia® (Sedana Medical) gas monitor. Data were obtained from patients' medical records.

## Results

We included 61 adult patients who were admitted from April 2012 to November 2014. Mean age was 62.6 ± 14.9 years, 39 (63.9%) were male. ICU mortality was 41%, hospital mortality was 43%. Mean duration of sevoflurane use was 3.56 ± 2.31 days. Admission diagnoses were: successful resuscitation after cardiac arrest (44.2%), sepsis (37.7%), cardiogenic shock (4.9%), pancreatitis (3.3%) and liver failure, acute exacerbation of COPD, asthma, tetanus and intracerebral hemorrhage (1.6% each). During treatment with sevoflurane, no patients developed malignant hyperthermia, new hyperkalemia or QT prolongation. In three (4.9%) patients, worsening liver function tests prompted sevoflurane discontinuation. Ischemic hepatitis was considered an alternative in all three patients. Seven (11.4%) patients developed renal failure while receiving sevoflurane. Sevoflurane was continued in all patients and renal failure was attributed to alternative diagnoses. No self-extubations were recorded. In seven (11.4%) patients, sevoflurane was discontinued because of worsening ventilation. In six (9.8%) patients, unexpected awakening occurred. Eight patients (13.1%) had symptoms of delirium after sevoflurane inhalations were concluded.

## Conclusion

We identified sevoflurane as an appropriate sedation agent in a diverse group of patients. Sevoflurane advantages over intravenous sedation could be more pronounced in some patient groups (for example, successful resuscitation after cardiac arrest). The safety profile of sevoflurane sedation was comparable with intravenous sedation [1].
